# Hygienic Heresy

**Published:** 1889-02

**Authors:** S. H. Preston


### HYGIENIC HERESY.

BY S. H. PRESTON.

Physiology is frequently forced to face very stunning and unassailable facts. After ascertaining and asserting the conditions of good health and long life, it is confronted by the fact, that folks who most faithfully comply with those conditions fall the first victims to disease and death.

Again, it appears often that those who pursue the most unhygienic and vicious course of life are the healthiest and the longest livers. The man who daily and industriously violates every precept of physiology, as though he sought suicide thereby, sometimes proves to be the soundest and the strongest man in town; while some frail and constantly complaining woman lives to be the oldest inhabitant. Every instance I have ever known of remarkable longevity has been an inveterate user of tobacco or intoxicating drink, or a person addicted to pernicious conduct of some kind.

The common cry of every health hobbyist is for pure air. That is said to be the sole safeguard against consumption. Well, the purest air in America, is admitted to be up on the hills of New England. Accordingly, there should be no consumption there. But what is the fact? Why, consumption is the prevalent complaint, and the old residents are dying off with it. The foulest air on this continent, that most loaded with miasm and impurities, is undoubtedly among the marsh lands of Kentucky. Now, a case of consumption has never been known among the natives who breathe this noxious air, and who subsist on hog and hominy and corn-juice whiskey. And weak-lunged persons who, by advice of physicians, go away in quest of healthy air, usually return in charge of the undertaker. Hygienists hold that there is nothing so deadly as to sleep with covered head in a closed room. Ventilate, open the windows, saturate the air with ozone, say they. But the dog, guided by the promptings of nature, when it lies down to sleep, gets its head under the stove or into some close and stifling corner, and covers its nose with its paws.

Pure drinking water is another physiological prerequisite of health. All the resources of science and invention, have been utilized in filtering and demineralizing and medicating water, so it will be fit to drink. And the more the water is purified and improved, the more dyspepsia and kidney complaints prevail. Some animals avoid the clear cold water of the moss-rocked spring and seek the dirty water of the distant river, while tribes in sections where the only obtainable water is so thick with impurities that it is more meat than drink, preserve perfect health and know nothing of Bright's disease.

A few years ago there were so many instances of extreme age reported from a certain district in England, that a medical commission was sent there to ascertain the conditions of life that contributed to such longevity. The investigators were astounded at the state of affairs they found, and their statement of facts seems incredible. The place was a little smoky manufacturing settlement, surrounded by marshes and stagnant water, without drainage or the common sanitary accessories of civilization. Both air and drinking water were as bad as they could be, and it appeared impossible that any resident could long retain health or life.

Here in New York the healthiest, handsomest, and happiest young ones will be found, squalid and neglected, in pestiferous slums and hell's kitchen and battle alley, while if you would see white, weazen-faced little things, looking like blighted buds of humanity, go to Murray Hill and Madison Avenue, where center the choicest conditions of health that affluence can procure.

Now these are facts that no hygienist can ignore, and that medical science must ere long explain. Perhaps it may yet appear that all our physiological notions are falsely founded, and in practice induce more disease than they prevent. Absolutely pure air may be found to be as fatal as sewer gas, and perfectly pure water worse than whiskey.

The order of nature is one of perfect adaptation. And may be in her economy, man's organization still requires a certain proportion of impurities, and that to abruptly alter his mode of living, even to improve it, would be like putting a salt-water ash into a mountain brook. Were there scientific salmon we might suppose them saying "See here, this salt is a mineral poison and is killing us off. We must get into fresh water as fast as we can." And were a pollywog to turn physiologist he would say to his associates, "Why, this is a stagnant, stinking puddle. To save our health we must skip for Saratoga."

Nature has undoubtedly adapted the surroundings of man to his conditions, and it is to be presumed that he is not yet enough angelized for perfectly pure food, air and water. Were it possible for him to procure these in his present state of physical imperfection, they would probably cause him to become an angel prematurely.

